# Brain-Derived Microparticles (BDMPs) Contribute to Neuroinflammation and Lactadherin Reduces BDMP Induced Neuroinflammation and Improves Outcome After Stroke

**DOI:** 10.3389/fimmu.2019.02747

**Published:** 2019-11-26

**Authors:** Zhili Chen, Michael Chopp, Alex Zacharek, Wei Li, Poornima Venkat, Fenjie Wang, Julie Landschoot-Ward, Jieli Chen

**Affiliations:** ^1^Department of Neurology, Henry Ford Hospital, Detroit, MI, United States; ^2^Department of Physics, Oakland University, Rochester, MI, United States

**Keywords:** brain-derived microparticles, stroke, neuroinflammation, Lactadherin, therapy

## Abstract

Microparticles (MPs, ~size between 0.1 and 1 mm) are lipid encased containers derived from intact cells which contain antigen from the parent cells. MPs are involved in intercellular communication and regulate inflammation. Stroke increases secretion of brain derived MP (BDMP) which activate macrophages/microglia and induce neuroinflammation. Lactadherin (Milk fat globule–EGF factor-8) binds to anionic phospholipids and extracellular matrices, promotes apoptotic cell clearance and limits pathogenic antigen cross presentation. In this study, we investigate whether BDMP affects stroke-induced neuroinflammation and whether Lactadherin treatment reduces stroke initiated BDMP-induced neuroinflammation, thereby improving functional outcome after stroke. Middle aged (8–9 months old) male C57BL/6J mice were subjected to distal middle cerebral artery occlusion (dMCAo) stroke, and BDMPs were extracted from ischemic brain 24 h after dMCAo by ultracentrifugation. Adult male C57BL/6J mice were subjected to dMCAo and treated via tail vein injection at 3 h after stroke with: (A) +PBS (*n* = 5/group); (B) +BDMPs (1.5 × 10^8^, *n* = 6/group); (C) +Lactadherin (400 μg/kg, *n* = 5/group); (D) +*BDMP*+Lactadherin (*n* = 6/group). A battery of neurological function tests were performed and mice sacrificed for immunostaining at 14 days after stroke. Blood plasma was used for Western blot assay. Our data indicate: (1) treatment of Stroke with BDMP significantly increases lesion volume, neurological deficits, blood brain barrier (BBB) leakage, microglial activation, inflammatory cell infiltration (CD45, microglia/macrophages, and neutrophils) into brain, inflammatory factor (TNFα, IL6, and IL1β) expression in brain, increases axon/white matter (WM) damage identified by decreased axon and myelin density, and increases inflammatory factor expression in the plasma when compared to PBS treated stroke mice; (2) when compared to PBS and BDMP treated stroke mice, Lactadherin and BDMP+Lactadherin treatment significantly improves neurological outcome, and decreases lesion volume, BBB leakage, axon/WM injury, inflammatory cell infiltration and inflammatory factor expression in the ischemic brain, respectively. Lactadherin treatment significantly increases anti-inflammatory factor (IL10) expression in ischemic brain and decreases IL1β expression in plasma compared to PBS and BDMP treated stroke mice, respectively. BDMP increases neuroinflammation and aggravates ischemic brain damage after stroke. Thus, Lactadherin exerts anti-inflammatory effects and improves the clearance of MPs to reduce stroke and BDMP induced neurological deficits.

## Introduction

Stroke is a leading cause of mortality and severe long-term disability worldwide (1). In addition to the challenges of day-to-day activities due to neurological deficits, stroke is also a huge economic burden to patients and caregivers (2). Despite extensive research in the past few decades, there are few treatment options for stroke and stroke remains a global health concern (3). Therefore, there is an urgent need to identify effective treatments for stroke.

Microparticles (MPs, size between 0.1 and 1 mm) are a class of small membrane-bound vesicles that are shed from the cell membrane (4). MPs can be released into the blood and body fluids by cells during activation, necrosis or apoptosis (5). A massive amount of MPs are released into the circulation after acute brain injury (6–9). These MPs are broadly classified into: (1) circulating MPs that are derived from endothelial cells, platelets, leukocytes, and (2) brain derived MPs (BDMPs) (10, 11). Both types of MPs may be involved in disease development. Circulating MPs have been investigated as potential biomarkers for a variety of neurological disorders including ischemic cerebrovascular accidents, transient ischemic attacks, multiple sclerosis, and cerebral malaria (12). MPs play a key role in peripheral inflammatory progression, thrombosis, endothelial dysfunction, and angiogenesis (13–15). Microglial/macrophage-derived MPs and BDMPs can increase brain inflammation in normal mice (16, 17). MPs increase the permeability of blood-brain barrier (BBB) (18) and BDMPs can migrate through the disrupted endothelial barrier (19). These observations led us to hypothesize that BDMPs contribute to neuroinflammation after stroke, and thereby increased BDMP clearance would improve neurological recovery.

Lactadherin (milk fat globule-epidermal growth factor 8, Lactadherin) is a multifunctional glycoprotein originally identified as part of the milk fat globule membrane. Lactadherin couples apoptotic cells with monocytes/macrophages to facilitate phagocytosis (20–22) and clearance of apoptotic cells, and regulates immune response after stroke (23–26). In this study, we are the first to investigate that BDMPs contribute to neuroinflammation after stroke, while Lactadherin promotes the clearance of BDMPs and reduces inflammation and thereby improves ischemic stroke outcome.

## Materials and Methods

All experiments were conducted in accordance with the standard and procedures of the American Council on Animal Care and Institutional Animal Care and Use Committee of Henry Ford Health System.

### Experimental Groups

Middle aged (8–9 months) male C57/BL6 mice (Jackson Laboratory) were subjected to distal middle cerebral artery occlusion (dMCAo) and randomly divided into the following treatment groups: (1) PBS (*n* = 5); (2) +BDMPs (1.5×10^8^, *n* = 6); (3) +Lactadherin (400 μg/kg, tail vein injection, Hematologic Technologies, Essex Junction, VT, *n* = 5); (4) +BDMP+Lactadherin (*n* = 6); (5) Sham control (*n* = 6); (6) Sham+BDMP (*n* = 5). Treatments were administered via tail vein injection at 3 h after stroke.

### Photothrombotic Stroke Model

To generate a consistent infarct volume, focal cortical ischemia was induced by photothrombosis of the cortical microvessels, as previously described (27). Briefly, mice were anesthetized with chloral hydrate (0.3 mg/kg, i.p). A light sensitive dye, Rose Bengal (100 μ*l*/ <25 g, 150 μl /25–40 g, 10 mg/ml solution in saline; SigmaAldrich, St Louis, MO) was administered i.p. A midline incision of the scalp was performed to expose the skull. The skull was covered by a roundabout black rubber to expose the area of 0.7–2.7 mm right to the midline, −2.5–1 mm rostral to the bregma. The brain was illuminated for 15 min through the exposed skull with a fiber-optic bundle of a cold light source (KL 1600 LED; Schott, Mainz, Germany) filtered with a green filter. The scalp incision was sutured and mice returned to home cages to awaken. Sham control mice were subjected to the same surgical protocol as above, but without injection of Rose Bengal.

### Neurological Function Test

To assess neurological functional outcome, a battery of functional tests including a modified neurological severity score (mNSS) test (28) and foot-fault test (29) were performed before dMCAo and after dMCAo on days 1, 3, 7 and 14 by an investigator who was blinded to the experimental groups.

### BDMP Isolation

BDMP isolation was performed following previously published methods (7, 9, 19). Briefly, the ischemic brain was harvested 24 h after stroke and quickly frozen in liquid nitrogen. To isolate BDMPs, brain was rapidly thawed at room temperature and homogenized in 1 ml of PBS using a glass Dounce homogenizer (Fisher Scientific Co., Federal Way, WA). The homogenate was centrifuged at 1,500 g for 20 min at 4°C to remove intact cells. The supernatant was centrifuged at 13,000 g for 2 min at 4°C to remove large cellular debris, and then centrifuged twice at 100,000 g for 1 h at 4°C, using a TLA-100.4 rotor (Beckman Coulter, Miami, FL). The pellet was resuspended in 500 μl of PBS. MPs were quantified by flow cytometry in a time fixed mode in the presence of counting beads (Spherotech, Lake Forest, IL). Megamix microbeads [0.5, 0.9 and 3 μm (Biocytex, Marseille, France)] were used to gate microparticles based on the particle size.

### Immunohistochemistry

All animals were euthanized 14 days after stroke and transcardially perfused with cold 0.9% saline. Brains were isolated and immersion fixed in 4% paraformaldehyde before being embedded in paraffin. A series brain coronal sections (6 μm thick) were cut from the center of the lesion (bregma −2.5 mm~+1 mm). Hematoxylin & eosin (H&E) stain was used to identify the lesion volume. All immunostainings were performed at 14 days after stroke. Antibodies against CD45 (a marker for lymphocytes, 1:500, Abcam), IBA-1 (a marker for microglia/macrophages, 1:1,000, Abcam), myelin basic protein (MBP, a marker for myelin, 1:300, Dako), CD31 (a marker for vessel, 1:200, Dako), Antibody against albumin (Albumin-FITC, 1:500, Abcam), NeuN (a marker for neuronal,1:50, Millepore), Myelo-peroxidase (MPO, a marker for neutrophil, 1:200, Dako), Interleukin 1β (IL1β,1:200, Abcam), Interleukin 6 (IL6,1:200, Abcam), Tumor necrosis factor (TNFα,1;200, Abcam), and Interleukin 10 (IL10, 1:200, Abcam) were employed. Bielschowsky silver (BS) staining was used to demonstrate axons and luxol fast blue (LFB) staining was used to demonstrate myelin. Three slides from each brain, with each slide containing five fields from cortex and striatum of the ischemic border zone (IBZ) were digitized under 20× objective (Olympus BX40) using a microscope (Sony DXC-970MD). The number of positive cells of neuronal, CD31, CD45, IBA-1, neutrophils, IL6, IL1β, TNFα, IL10, and the positive areas of Albumin, BS, LFB and MBP were calculated by Image Pro Plus 6.0. Immunohistochemical analysis was performed by an investigator who was blinded to the experimental groups.

### Lesion Volume Measurement

Seven coronal sections of tissue were stained with hematoxylin and eosin (H&E) for lesion volume calculation. Data are presented as a percentage of lesion compared with the contralateral hemisphere (30). Measurements were performed by an investigator who was blinded to the experimental groups.

### Western Blot

Equal amounts of plasma samples were subjected to Western blot analysis, as previously described (31). Protein concentration was measured using BCA Protein Assay Kit (Thermo Fisher Scientific, USA). Forty micrograms of protein/lane in a 10% SDS PAGE precast gel (Invitrogen). Gel was transferred using an iBlot transfer system (Invitrogen) following standard protocol. Nitrocellulose membrane was blocked in 2% I-Block (Applied Biosystems) in 1× TBS-T for 1 h. Primary antibody against IL1β (1:1,000, Abcam, Cambridge, MA, USA) was employed. Anti-β-actin (1:10,000, Abcam, Cambridge, MA, USA) was employed for control measurements. Secondary antibody was added at 1:5,000 dilution in 2% I-Block in 1× TBS-T on a room temperature shaker for 1 h. The membranes were then developed using a FluorChem E Imager system (ProteinSimple) exposing them for 1–30 min depending on the intensity of the band. Bands were analyzed using ImageJ.

### Statistical Analysis

Repeated measure analysis of variance (ANCOVA) was used to study the group differences in mNSS and foot-fault function tests over time (time points: 1, 3, 7, and 14 days). The one-way analysis of variance (ANOVA) was used to evaluate immunostaining and Western blot. All data are presented as mean ± SE.

## Results

### BDMPs Do Not Induce Neurological Deficit and Brain Damage in Wild Type Control Mice

First, we tested whether BDMPs induce brain damage and neurological function deficit in non-stroke sham control mice. We found that (Supplementary Figure 1) injection of BDMPs into sham non-stroke (Sham+BDMP) mice did not induce neurological functional deficits (Supplementary Figures 1A,B), and no axon/white matter damage was evident in the brain tissue identified by BS and LFB staining when compared to sham control mice (Supplementary Figures 1C,D). Injection of BDMP into sham non-stroke (Sham+BDMP) mice did not induce leukocyte (CD45, Supplementary Figure 1E) infiltration or increase microglial activation (IBA-1, Supplementary Figure 1F) in brain when compared to sham control mice.

### BDMPs Aggravate and Lactadherin Treatment Attenuates Neurological Impairment and Lesion Volume After Stroke in Mice

**Figure 1:**
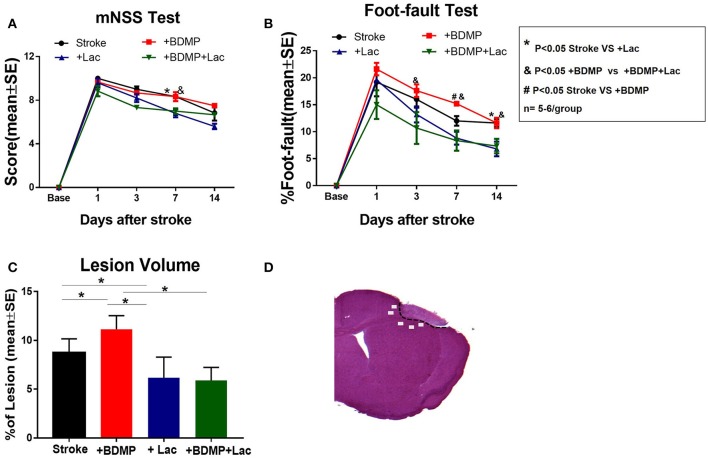
BDMPs aggravate and Lactadherin attenuates neurological impairment and lesion volume after stroke in mice. **(A)** mNSS and **(B)** Foot-fault tests were performed at 1, 3, 7, and 14 days after stroke. **(C)** Stroke lesion volume was calculated by H&E stains. *n* = 5–6/group. **(D)** Immunostaining measurement from cortex and striatum of the selected five fields in the ischemic border zone (IBZ). *P < 0.05 Stroke vs. Stroke+lac; ^&^*P* < 0.05 Stroke+BDMP vs. Stroke+BDMP+lac; ^#^*P* < 0.05 Stroke vs. Stroke+BDMP; *n* = 5–6/group; Data are presented as mean ± SE.

To evaluate the effects of BDMPs and Lactadherin treatment on neurological function after stroke in mice, mNSS, and foot-fault tests were employed. Figures 1A,B shows that injection of BDMPs significantly aggravates neurological impairment in stroke mice when compared to PBS dMCAo control group. Injection of BDMPs significantly increases ischemic lesion volume in the brain identified by H&E staining (Figure 1C). Administration of Lactadherin together with PBS or BDMPs significantly improves neurological function when compared to stroke mice receiving PBS or BDMPs alone, respectively (Figures 1A,B). Administration of Lactadherin together with PBS or BDMPs significantly decreases ischemic lesion volume when compared to stroke mice receiving PBS or BDMPs alone, respectively (Figure 1C).

### BDMP Aggravates and Lactadherin Attenuates BBB Leakage and Neuronal Loss While Increasing Vascular Density in Stroke Mice

**Figure 2:**
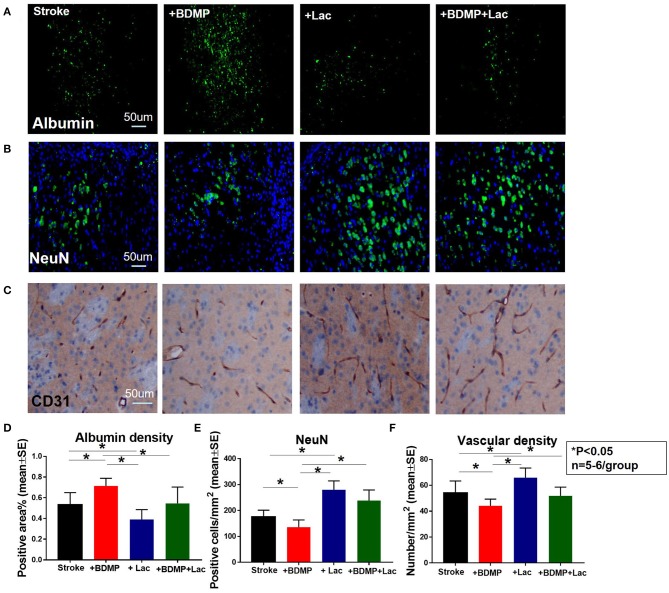
Lactadherin treatment significantly attenuates BBB leakage and neuronal loss induced by injection of BDMPs in stroke mice, while promoting vascular density. **(A)** FITC-Albumin and **(B)** neuron staining indicate that Lactadherin treatment significantly reduces BDMPs induced BBB leakage and neuronal loss. **(C)** Lactadherin treatment significantly increases vascular density. *n* = 5–6/group. Scale bar, 50 μm. **(D–F)** Quantification data. *p < 0.05. Data are presented as mean ± SE.

To evaluate the effects of circulating BDMPs and Lactadherin on BBB integrity, vasculature and neuronal injury, we evaluated FITC-albumin, neuron and vascular density in the cortex and striatum of IBZ (Figure 1D). Figure 2 shows that BDMPs injection significantly increases BBB permeability, decreases vascular density and results in greater neuronal loss compared to PBS treated stroke mice. Lactadherin treatment significantly decreases BBB leakage, increases vascular density and attenuates neuronal loss compared to stroke mice treated with PBS or BDMPs, respectively (Figure 2).

### BDMPs Significantly Increase Axonal/WM Damage in Ischemic Brain While Lactadherin Treatment Significantly Promotes Axonal/WM Density After Stroke in Mice

**Figure 3:**
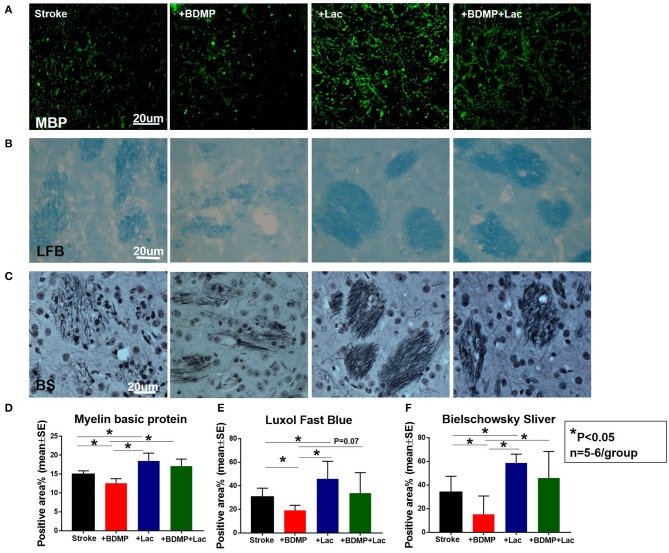
Lactadherin treatment significantly attenuates axonal/WM damage induced by BDMPs in stroke mice. **(A)** MBP, **(B)** Luxol fast blue, and **(C)** Bielschowsky sliver staining data indicate that Lactadherin treatment significantly increases axonal/WM density after injection of BDMPs in stroke mice, while BDMPs significantly increases axonal/WM damage. *n* = 5–6/group. Scale bar, 20 μm. **(D–F)** Quantification data. *p < 0.05. Data are presented as mean ± SE.

To test whether BDMPs aggravate and Lactadherin treatment reduces axonal/WM injury after ischemic stroke in mice, we employed MBP, BS, and LFB staining to quantify WM changes in the cortex and striatum of IBZ. Figure 3 shows that injection of BDMPs significantly decreases axon and myelin density in the IBZ compared to stroke group. Lactadherin treatment significantly increases axon and myelin density compared to stroke mice treated with PBS or BDMPs, respectively.

### BDMPs Significantly Increase and Lactadherin Treatment Significantly Decreases Neuroinflammation After Stroke in Mice

**Figure 4:**
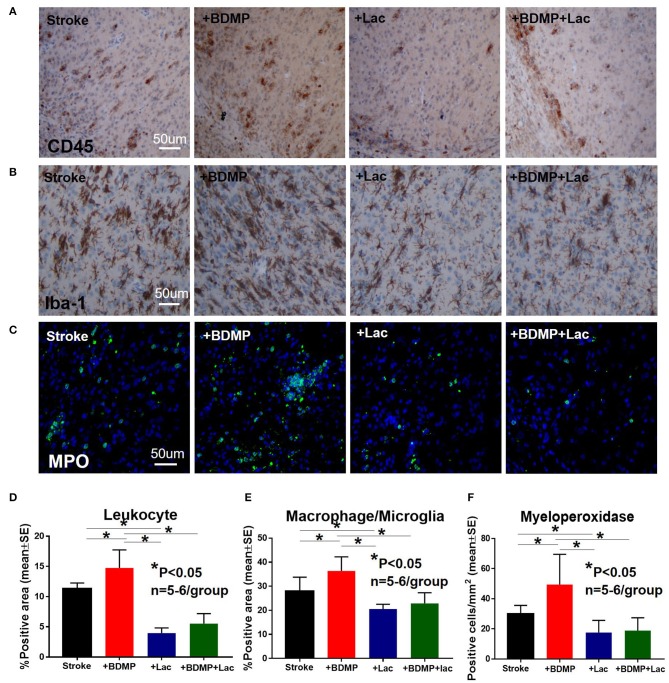
BDMPs significantly increase and Lactadherin treatment significantly decreases inflammatory cell infiltration after stroke in mice. BDMPs significantly increase: **(A)** leukocyte (stained by CD45), **(B)** microglia/macrophage and, **(C)** neutrophil infiltration in ischemic brain, while Lactadherin treatment significantly decreases infiltration of inflammatory cell. *n* = 5–6/group. Scale bar, 50 μm. **(D–F)** Quantification data. *p < 0.05. Data are presented as mean ± SE.

**Figure 5:**
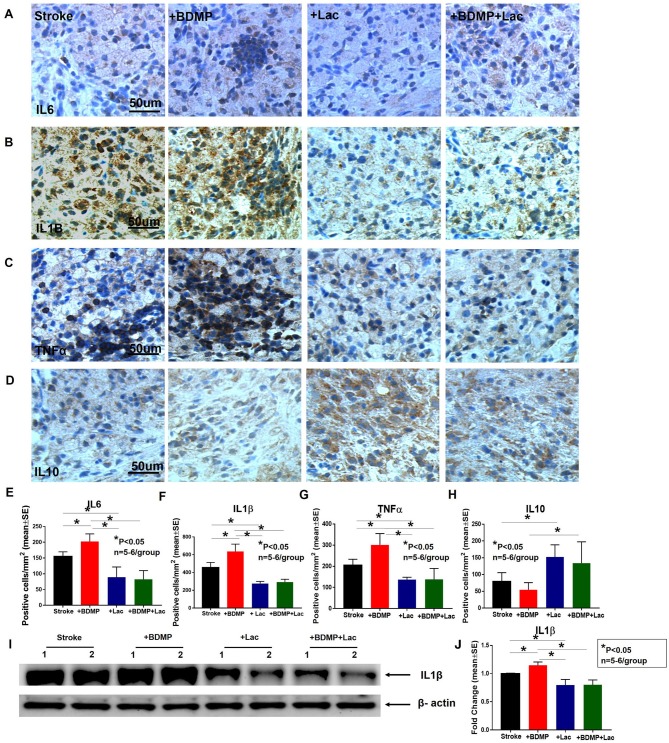
BDMPs significantly increase and Lactadherin treatment significantly decreases inflammatory factor expression in ischemic brain. BDMPs significantly increase: **(A)** IL6, **(B)** IL1β, and **(C)** TNFα expression in ischemic brain, while Lactadherin treatment significantly decreases inflammatory factor expression. **(D)** Lactadherin increases anti-inflammatory factor IL10 expression in brain after stroke. **(I,J)** The Western blot result shows that BDMPs significantly increase and Lactadherin treatment significantly decreases IL1β expression in circulation. *n* = 5–6/group. Scare bar, 50 μm. **(E–H)** Quantification data. *p < 0.05. Data are presented as mean ± SE.

To evaluate the inflammatory responses of BDMP injection and Lactadherin treatment in stroke mice, we measured the expression of leukocytes, microglia/macrophages, neutrophils, IL1β, IL6, and TNFα in the cortex and striatum of IBZ. As indicated in Figures 4, 5, injection of BDMPs after stroke significantly increases inflammatory cell and pro-inflammatory factor expression compared to stroke alone group. Lactadherin treatment significantly decreases inflammatory cell expression after stroke as well as attenuates BDMP induced neuroinflammation. In addition, Lactadherin treatment also significantly increases anti-inflammatory factor IL10 expression in IBZ compared to stroke mice treated with PBS or BDMPs, respectively (Figure 5). Figures 5I,J shows that BDMP injection significantly increases inflammatory factor IL1β expression in the circulation, while Lactadherin treatment significantly decreases IL1β expression in the circulation compared to stroke mice treated with PBS or BDMPs, respectively.

## Discussion

In this study, we demonstrate for the first time that BDMPs aggravate and Lactadherin attenuates stroke induced neurological deficits, BBB leakage, loss of vascular density, neuronal loss, axonal/WM injury and neuroinflammation after stroke in mice. These data suggest that neuroinflammation mediated by BDMPs may contribute to brain injury after stroke.

Extracellular MPs may play an important role in the pathological development and prognosis after stroke (8, 11, 32). MPs can induce neuronal damage and neurotoxicity (33, 34). Previous studies have reported that the injured brain releases BDMPs into the circulation (5, 7, 9, 16). Circulating MPs are lipid encased containers (sized 0.1–1.0 μm) that are shed from the plasma membrane of eukaryotic cells upon injury, activation, or apoptosis (35). Microparticles are vulnerable to degradation and clearance. Charoenviriyakul et al. reported that after intravenous injection different types of exosomes into mice, all the exosomes rapidly disappeared from the systemic circulation and were primarily localized to the liver (36). Exosomes can be taken up by macrophages and undergo clearance (37).Pharmacokinetic studies show that intravenously injected exosomes in mice were 10% of the initial injected amount at 4 h post injection (36). Clearance of all types of exosomes in macrophage-depleted mice was significantly delayed compared to that in non-macrophage depleted mice, indicating that macrophages play a key role in the clearance of exosomes from the blood circulation (36). The clearance of circulating microparticles involve direct receptor binding of liver or spleen phagocytes to phosphatidylserine or to opsonization proteins on the microparticles (38, 39). The routes of clearance microparticles by cells and organs include endocytosis (clathrin- and caveolin-dependent and lipid-raft-mediated), micropinocytosis, phagocytosis and membrane fusion (40). In addition, the rates of microparticle degradation and clearance vary and depend on the ways that cells interact with their environment (41). The pooled concentrations of total MP, i.e., BDMPs, platelet-derived MPs, endothelial-derived MPs, leukocyte-derived MPs, erythrocyte-derived MPs, and monocyte-derived MPs are significantly increased in ischemic stroke patients compared to non-cerebrovascular disease controls, all of which are associated with poor clinical outcome (7, 11, 42, 43). BDMPs can contribute to the progression of neuroinflammatory diseases and promote inflammatory activities, as well as promote the development and regeneration of the nervous system after stroke (44–46). Microglia-derived MPs and astrocyte-derived MPs contain and release the proinflammatory cytokine IL-1β, inflammasome components and MHCII proteins (47, 48). Previous studies have found that enriched MPs from activated microglia *in vitro* or from mice brain are sufficient to initiate neuroinflammation following intracortical injection in naïve animals (16, 17).

Lactadherin mediates cell-cell interactions and is involved in various physiological and pathophysiological functions including angiogenesis (49), fertilization (50), inflammation (23) and clearance of apoptotic cells (51). Several studies have confirmed the therapeutic effects of Lactadherin in stroke (23, 24, 26, 52). Lactadherin exerts neuroprotection against cerebral injury by suppressing inflammation, reducing neuronal cell death, promoting apoptotic cell clearance (26). Additionally, Lactadherin improves subarachnoid hemorrhage (SAH) outcome via anti-oxidation which may be dependent on integrin β3/ nuclear factor erythroid 2-related factor 2/HO pathway (24). MFG-E8 maintains a role in the association between brain microvessels and surrounding brain parenchyma (53). A recent study indicates that Lactadherin can couple apoptotic cells with monocytes/macrophages to facilitate phagocytosis and promote the clearance of BDMPs (19, 21, 22, 54). We employed Lactadherin treatment together with BDMP administration to detect whether Lactadherin can promote neurological recovery by clearing BDMPs and decrease BDMP-induced neuroinflammation. Our data indicate that circulating BDMPs released by injured brain aggravate stroke outcome as evidenced by worse neurological deficits and exacerbated neuronal loss and lesion volume, while Lactadherin treatment improves neurological function, attenuates neuronal loss, and decreases lesion volume after stroke.

Physiological responses after stroke include BBB break down, delayed angiogenesis and neuron death (55). Previous studies have shown that neuronal damage in the central nervous system (CNS) is correlated with blood-brain barrier (BBB) breakdown (56–58). BBB is important for maintaining stability of the brain microenvironment of brain. BBB disruption permits infiltration of peripheral immune cells into the parenchyma and increases vascular oxidative stress and neuroinflammation which play a critical role in neuronal loss (59–61). MPs are known to increase BBB permeability (18, 62, 63). Our data indicate that injection of BDMPs increase BBB leakage compared to the Stroke+PBS group, and Lactadherin treatment decreases BBB leakage which may contribute to improved neurological function in stroke mice. Post-ischemic angiogenesis has been widely associated with the recovery of blood flow in peri-infarct brain regions (64). The extent of angiogenesis plays a crucial role in long-term neurological function recovery (65–68). Our data show that injection of BDMPs reduces angiogenesis which may partially contribute to worse neurological function and increased infarction volume. Lactadherin treatment increases cerebral vascular density in the IBZ in stroke mice treated with PBS or BDMPs. Previous studies have demonstrated that MPs can induce neuronal damage and neurotoxicity (33, 34). Lactadherin can promote angiogenesis (49). Angiogenic vessels can increase cerebral blood flow in peri-infarcted brain regions, which may restore cellular metabolism in surviving neurons and promote neurogenesis (29). In our study, our data show that BDMPs increase neuronal loss in the IBZ, and Lactadherin treatment attenuates neuronal damage.

The white matter (WM) in the brain is highly susceptible to hypoxia and is injured following ischemic stroke (29). WM injury impairs neuronal connectivity and induces worse outcome after stroke (69, 70). WM promotes communication and sensory/motor reflex, which helps to restore lost nerve function and reduce symptoms of paralysis caused by stroke (29). Thus, WM function such as axonal regeneration and regrowth, axonal sprouting and remyelination in the peri-infarct region is critical for long term functional recovery (71). In this study, we found that injection of BDMPs after ischemic stroke decreases axon and myelin density in the IBZ while Lactadherin treatment significantly increases axon and myelin density in the IBZ of stroke mice treated with PBS and BDMPs, respectively.

Ischemic stroke is known to trigger complex systemic and local immune responses (72–74). However, the underlying mechanisms of post-stroke neuroinflammation are largely unclear (72–74). Neuroinflammation plays a critical role in WM damage, axonal degeneration and myelin breakdown (75). Microglia are activated within minutes after stroke onset and stimulate the production of inflammatory cytokines and promote leukocyte infiltration which exacerbate brain damage (76). Our data show that BDMPs increase the infiltration of leukocytes, microglia/macrophages and neutrophils, and the expression of immune factors IL1β, IL6, and TNF-α in the IBZ of stroke mice. The infiltration of immune cells and molecules may indirectly or directly increase BBB permeability and promote tissue damage after stroke (77). Infiltrating neutrophils and microglia/macrophages promote BBB breakdown, WM damage, vascular damage and contribute to poor stroke outcome (12, 78, 79). Pro-inflammatory cytokines such as TNFα, IL6, and IL1β induce BBB hyperpermeability (12) as well as induce WM injury (80–82). Lactadherin treatment significantly reduces systemic inflammatory response, decreases immune cell infiltration into the ischemic brain, decreases pro-inflammatory cytokine expression in the IBZ, and increases anti-inflammatory responses in stroke and BDMP treated stroke mice. Therefore, BDMPs may exert their adverse effects on stroke outcome by promoting inflammatory responses and Lactadherin treatment decreases BDMPs and regulates anti-inflammatory responses to improve stroke outcome in mice.

## Limitations

In this study we have demonstrated that BDMPs induce neuroinflammation and aggravate neurological impairment after stroke, and Lactadherin treatment improves stroke outcome by promoting clearance of MPs as well as by exerting anti-inflammatory effects. In this study, we selected MPs derived directly from the fresh brain tissue. Future studies are needed to test the effects of MPs derived from brain and their role in mediating neuroinflammation after stroke. The mechanisms of BDMP clearance by Lactadherin warrant investigation. Ischemic stroke can induce profound vascular, axon/WM damage. Vascular remodeling is a complex process that involves changes of structure and architecture of blood vessels via cell growth, death, migration, and degradation of the extracellular matrix (ECM) (83). Ischemic stroke induces axon/WM damage (84). In this study, we demonstrated Lactadherin regulation of vascular density and BBB leakage and axon/myelin density at 14 days after stroke. Additional studies on the temporal profiles of Lactadherin on neurovascular remodeling and neurological outcomes are warranted.

## Conclusions

In this study, we found that BDMPs increase neuroinflammation and exacerbate brain damage after stroke in adult mice. Lactadherin exerts anti-inflammatory effects, improves the clearance of BDMPs, and may be a therapeutic strategy to reduce stroke and BDMP induced neurological dysfunction.

## Data Availability Statement

All relevant data is contained within the manuscript.

## Ethics Statement

The animal study was reviewed and approved by Institutional Animal Care and Use Committee of Henry Ford Health System.

## Author Contributions

ZC performed experiments, analyzed data, and wrote the manuscript. MC was involved in experimental design and gave final approval of manuscript. PV performed experiments and wrote the manuscript. AZ, WL, FW, and JL-W performed experiments. JC was involved in experimental design, wrote the manuscript, analyzed data, and gave final approval of manuscript.

### Conflict of Interest

The authors declare that the research was conducted in the absence of any commercial or financial relationships that could be construed as a potential conflict of interest.

## Supplementary Material

Supplementary Figure 1 BDMPs do not induce brain damage in sham control mice. **(A)** mNSS and **(B)** Foot-fault test show that injection of BDMP into sham non-stroke (Sham+BDMP) mice did not induce neurological functional deficit. **(B)** Axon/white matter damage were not evident in brain tissue identified by BS **(C)** and LFB **(D)** staining in Sham+BDMP group when compared to sham control mice. Injection of BDMP did not induce leukocyte **(E)** infiltration or microglia **(F)** activation.
