# Supplementary figures and images for: Brain-Derived Microparticles (BDMPs) Contribute to Neuroinflammation and Lactadherin Reduces BDMP Induced Neuroinflammation and Improves Outcome After Stroke
Source: Front Immunol. 2019 Nov 26;10:2747. doi: 10.3389/fimmu.2019.02747 (PMC6968774; doi:10.3389/fimmu.2019.02747)

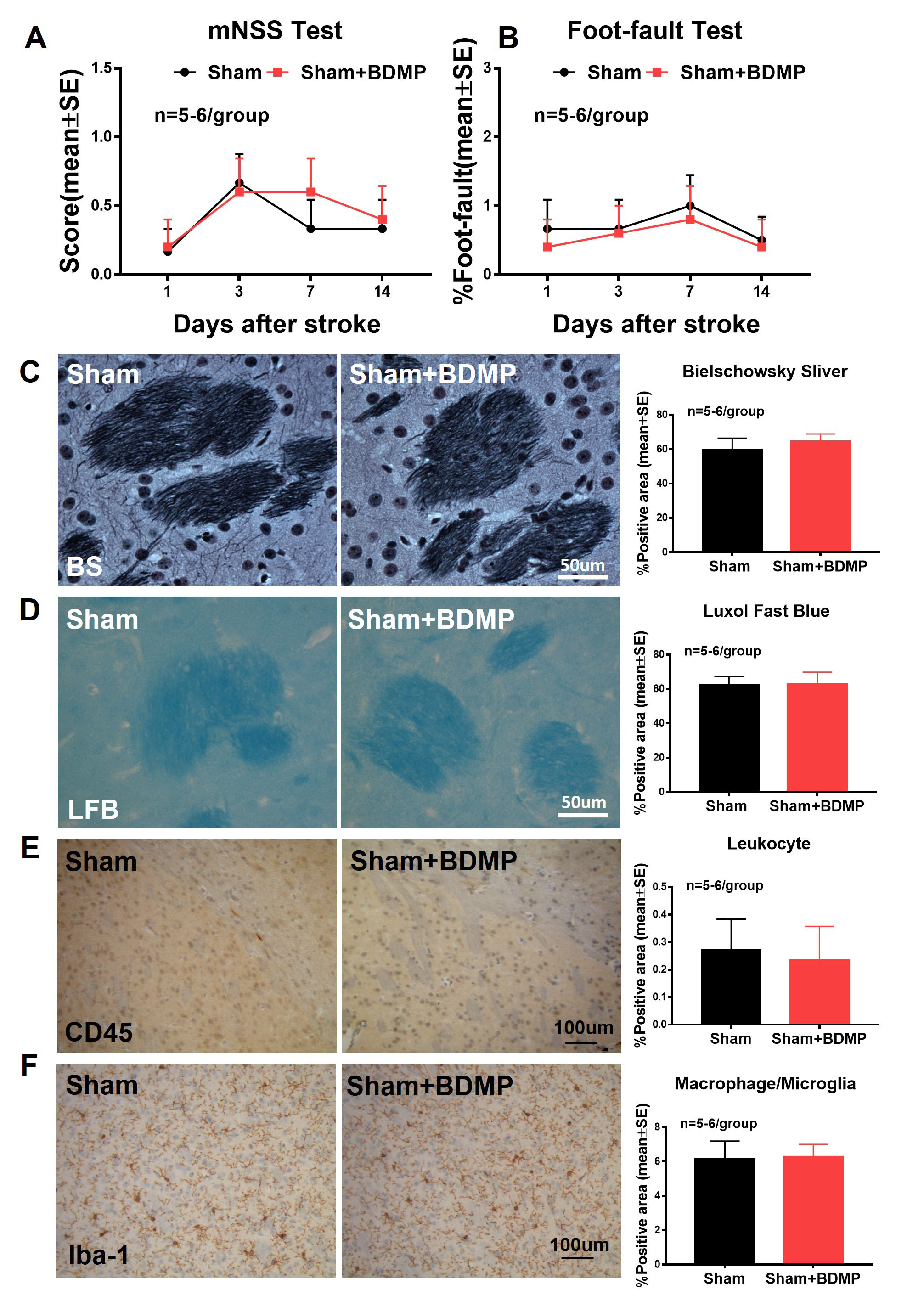

Supplement: Supplementary Figure 1 — BDMPs do not induce brain damage in sham control mice. (A) mNSS and (B) Foot-fault test show that injection of BDMP into sham non-stroke (Sham+BDMP) mice did not induce neurological functional deficit. (B) Axon/white matter damage were not evident in brain tissue identified by BS (C) and LFB (D) staining in Sham+BDMP group when compared to sham control mice. Injection of BDMP did not induce leukocyte (E) infiltration or microglia (F) activation. [file Image_1.JPEG]
